# Preclinical Pharmacokinetics of C118P, a Novel Prodrug of Microtubules Inhibitor and Its Metabolite C118 in Mice, Rats, and Dogs

**DOI:** 10.3390/molecules23112883

**Published:** 2018-11-05

**Authors:** Cang Zhang, Xiaolan Zhang, Guangji Wang, Ying Peng, Xueyuan Zhang, Hui Wu, Boyang Yu, Jianguo Sun

**Affiliations:** 1 School of Traditional Chinese Pharmacy, China Pharmaceutical University, Nanjing 211198, China; zhangcang@sanhome.com; 2 Nanjing Sanhome Pharmaceutical Co. Ltd., Nanjing 211135, China; 3 Key Lab of Drug Metabolism and Pharmacokinetics, State Key Laboratory of Natural Medicines, China Pharmaceutical University, Nanjing 210009, China; zhangxiaolan735@163.com (X.Z.); guangjiwang@hotmail.com (G.W.); pengy2014@126.com (Y.P.); xueyuanzh@yeah.net (X.Z.); wuhui0922@hotmail.com (H.W.)

**Keywords:** C118P, microtubules inhibitor, prodrug, pharmacokinetics, simplified tandem two-compartmental model

## Abstract

C118P, a phosphate prodrug of C118, which is a novel microtubule protein inhibitor, is currently under Phase I clinical development in China for treating ovarian cancer and lung cancer. The preclinical pharmacokinetics of prodrug C118P and its metabolite C118 were extensively characterized in vivo in mice, rats, and dogs and in vitro to support the further development of C118P. The preclinical tissue distribution and excretion were investigated in rats. Plasma protein binding in mice, rat, and human, and hepatic microsomal metabolic stability in mice, rat, dog, monkey, and human, were also evaluated. The (AUC_0-inf_) and C_30s_ of C118P at 50 mg/kg in rats and 6 mg/kg in dogs, and the C_2min_ of C118 at 6 mg/kg in dogs increased less than the dosage increase, suggested nonlinear pharmacokinetic occurred at high dose. As a prodrug, C118P can be quickly hydrolyzed into C118 after an intravenous administration. The unbound C118 in plasma is slightly higher than C118P. C118P can hardly penetrate the tissue, while C118 can distribute widely into tissues. In tumor-bearing nude mice, the concentration of C118 is high in lung, ovary, and tumor, with an extended half-life in tumor. C118P is a promising candidate prodrug for further clinical development.

## 1. Introduction

Cancer is the most important cause of death in China and has been a major public health problem, with increasing incidence and mortality. An estimated 4,292,000 new cancer cases and 2,814,000 cancer deaths occurred in China in 2015, with lung cancer being the most common incident cancer and the leading cause of cancer death [1]. In 1971, Folkman proposed the important role of angiogenesis in tumor growth and metastasis [2]. Tumor growth, invasion, metastasis and prognosis are related to tumor angiogenesis [3,4,5,6,7]. There are two types of therapeutic drugs for tumor blood vessels: anti-angiogenesis (VTA) agents and VDAs (vascular disrupting agents) mainly belonging to microtubule protein inhibitors which selectively destroy the tumor vascular system [8,9]. Acute administration of VDAs yields an immediate vascular collapse in contrast to angiogenesis inhibitors, which typically require multiple drug administration. VDAs can also increase vascular permeability, interstitial pressure causing plasma leakage, and reduce blood vessel diameter, blood flow and, therefore, vascular shutdown [10].

**Figure 1:**
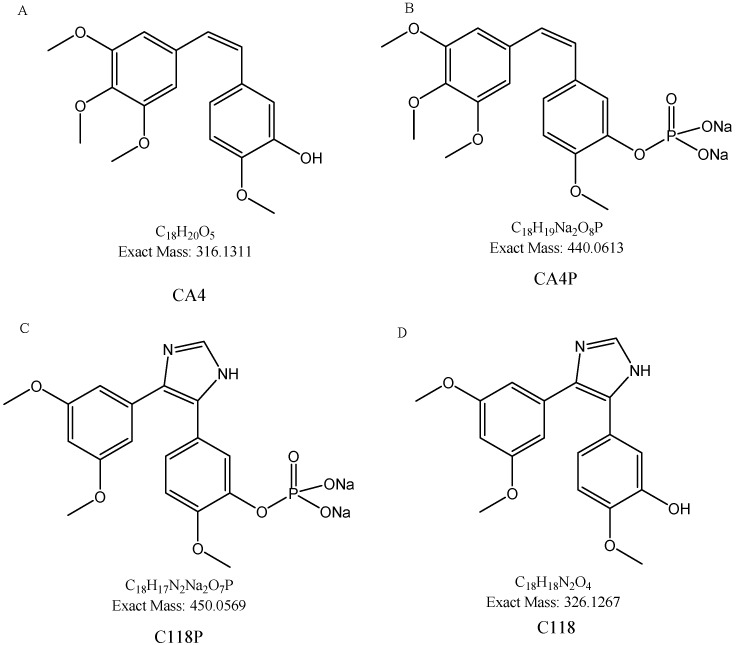
Chemical structure of CA4 (**A**), CA4P (**B**), C118P (**C**) and C118 (**D**).

Combretastain A4 (CA4, Figure 1A), isolated from the African bush willow *Combretum caffrum*, is the most potent naturally occurring combretastatins in regards to tubulin binding ability. Its prodrug CA4 phosphate (CA4P, Figure 1B) can inhibit tumor blood flow at far low concentration than the clinical maximal tolerated dose. CA4P can block new vessel formation and subsequently decrease cell migration and metastasis [10]. CA4P is mainly applied to thyroid cancer, ovarian cancer, non-small cell lung cancer and is currently in phase II study [11,12].

C118P (Figure 1C), an analog of CA4P, was developed by Nanjing Sanhome Pharmaceutical Co. Ltd. (Nanjing, China) as a phosphate prodrug of C118 (Figure 1D), and is currently under Phase I clinical trial in China for the treatment of solid tumors. The preliminary study showed that C118P displayed good inhibitory activities against ovarian cancer and lung cancer. Furthermore, the structure design of C118 avoids the potential generation of quinone metabolites in vivo. The efficacy and safety after structure modification were supposed to be improved.

In this study, the preclinical plasma pharmacokinetic (PK) profiles of C118P and C118 in mice, rats and dogs, the preclinical tissue distribution and excretion in mice and rats, were investigated after a single intravenous administration. The in vitro PK profiles of C118P and C118, including plasma protein binding and hepatic microsomal metabolic stability in different species, were also evaluated.

## 2. Results and Discussion

### 2.1. Plasma Protein Binding and Whole Blood/Plasma Ratio

Both C118P and C118 were stable in the plasma with EDTA as anticoagulant. The non-specific binding to the ultrafilter membrane was negligible. The extent of plasma protein binding was reported as a fraction unbound (fu, %) value which was calculated using the formula fu = PF/TC × 100%, where TC is test compound (C118P or C118) concentration in sample before ultrafiltration, and PF is the test compound concentration in the ultrafiltrate (Table 1).

The whole blood/plasma ratio in rat was 0.80 for C118P and 1.05 for C118, respectively. C118P and C118 can distribute into blood cell since the ratio is significantly higher than 0.55.

### 2.2. In Vitro Metabolism by Liver Microsomes

C118P was quite stable in liver microsomes of human, mice, rat, and monkey and was not stable in dog microsomes (Figure S1). The in vitro liver microsomal metabolism of C118 in various species, as measured by the disappearance of C118, is summarized in Table 2. The CLint, in vitro of C118, was 44.7, 67.0, 9.9, 318.6, and 58.2 μL·min^−1^·mg^−1^ protein in mice, rat, dog, monkey, and human liver microsomes, respectively. The rate of C118 metabolism in microsomes followed the order monkey > rat > human > mice > dog. Since the C118 is mainly metabolized in liver and excreted in bile, CL_H_ is supposed to be near CL. Compared with the observed CL, mouse and rat were best predicted with the accuracy within 2 folds. The prediction of dog is less accurate with accuracy larger than three-fold (Table 2).

### 2.3. PK in Mice, Rats, and Dogs

**Figure 2:**
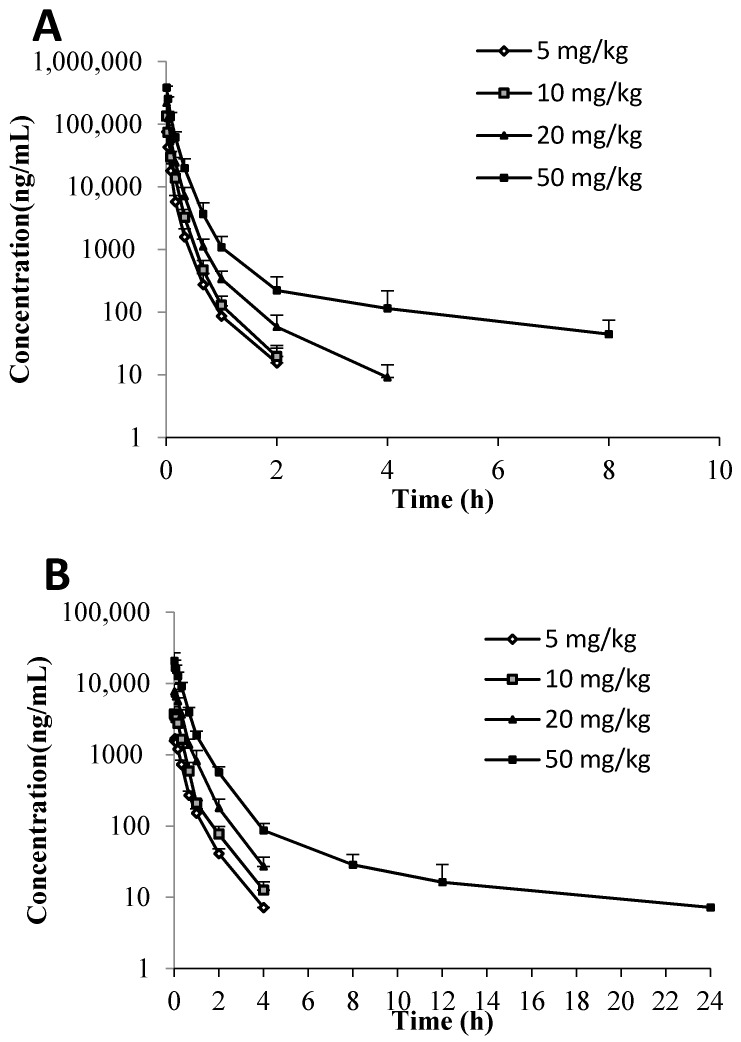

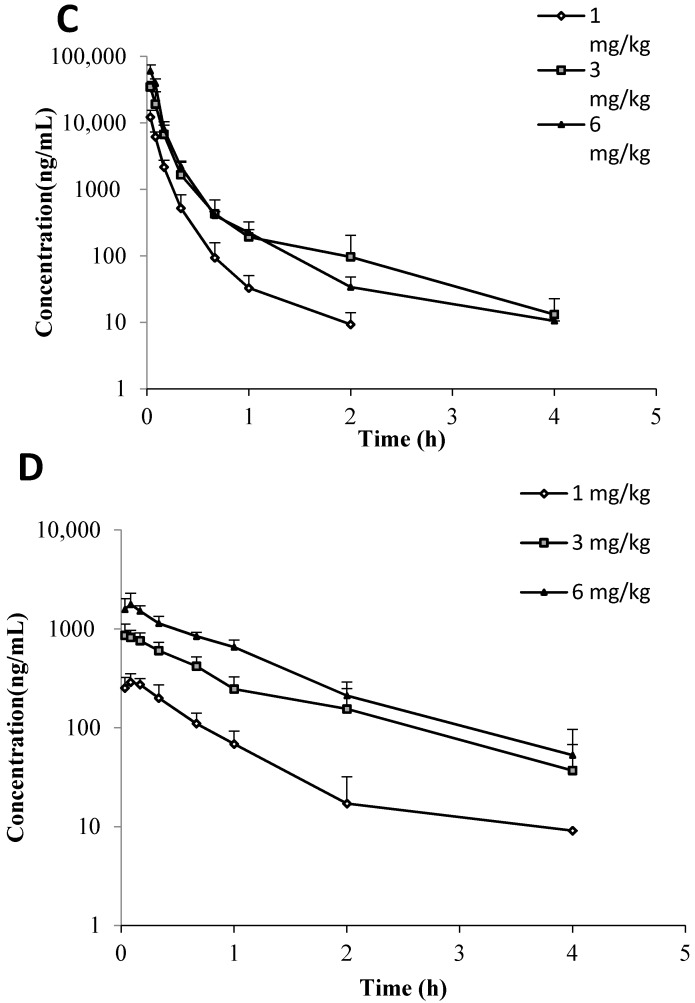
Plasma-concentration-versus-time profiles of C118P in rats (**A**), C118 in rats (**B**), C118P in dogs (**C**), and C118 in dogs (**D**) after *i.v.* injection of C118P. Doses were 5, 10, 20, and 50 mg/kg in rats and 1, 3, 6 mg/kg in dogs, respectively. Data are presented as the arithmetic mean ± SD (*n* = 6). Data are not illustrated in the figure due to levels below the lower limit of quantification (LLOQ, 5 ng/mL).

Both C118P and C118 were detectable in mice, rat, and dog plasma after *i.v.* injection of C118P. The plasma-concentration versus time profiles of C118P and C118 are shown in Figure 2. The PK parameters of C118P and C118 calculated from their concentrations in mice, rat, and dog plasma are presented in Table 3. The exposure (AUC_0-inf_) and C30s of C118P increased proportionally to the dose in the range of 5–20 mg/kg in rats and 1–3 mg/kg in dogs. The (AUC_0-inf_) and C30s of C118 increased proportionally to the dose in the range of 5–50 mg/kg in rats and 1–3 mg/kg in dogs. The (AUC_0-inf_) and C_30s_ of C118P at 50 mg/kg in rats and 6 mg/kg in dogs increased less than the dosage increase. The C_2min_ of C118 at 6 mg/kg in dogs increased less than the dosage increase, which suggested nonlinear pharmacokinetics occurred at high dose.

### 2.4. Tissue Distribution in Rats and Tumor Bearing Mice

**Figure 3:**
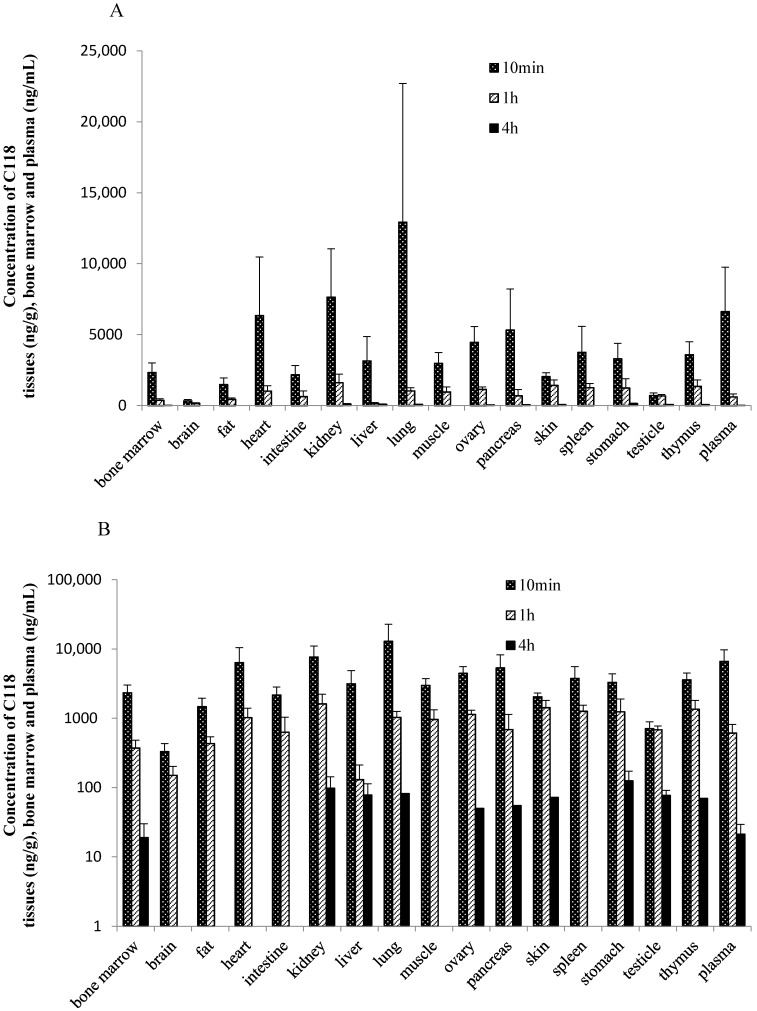
C118 concentration (ng/g, ng/mL) in rat tissues at 10 min, 1 h and 4 h after *i.v.* administration of C118P at 20 mg/kg. Data are presented as the arithmetic mean ± SD (*n* = 6). (**A**) The normal scale; (**B**) the semi-logarithm scale.

The tissue concentration of C118P in rats below or slightly higher than the LLOQ (50 ng/g for tissues) at 10 min, 1 h, and 4 h after intravenous administration 20 mg/kg of C118P, indicating that C118P can rarely distribute to tissues. In contrast to C118P, C118 was widely distributed in the tissues, namely lung, kidney, heart, pancreas, ovary, spleen, thymus, muscle, liver, stomach, bone marrow, small intestine, skin, fat, testicle, and brain listed in Figure 3. The highest tissue concentration of C118 was observed in the lung (12,921 ± 9785 ng/g tissue weight), followed by the kidney (7639 ± 3407 ng/g tissue weight) and heart (6335 ± 4130 ng/g tissue weight) at 10 min after dosing. The concentration of C118 was below or slightly higher than LLOQ (50 ng/g for tissues) at 4 h after dosing in most tissues.

The distribution of C118P and C118 in tissues of tumor bearing mice was studied and the results were showed in Table 4. Drug distribution in mice showed that C118P can hardly distribute into tissues. C118 can wildly distribute into different tissues. C118 was cleared from the tumor tissue much slower than other tissues as well as plasma.

### 2.5. Excretion in Rats

**Figure 4:**
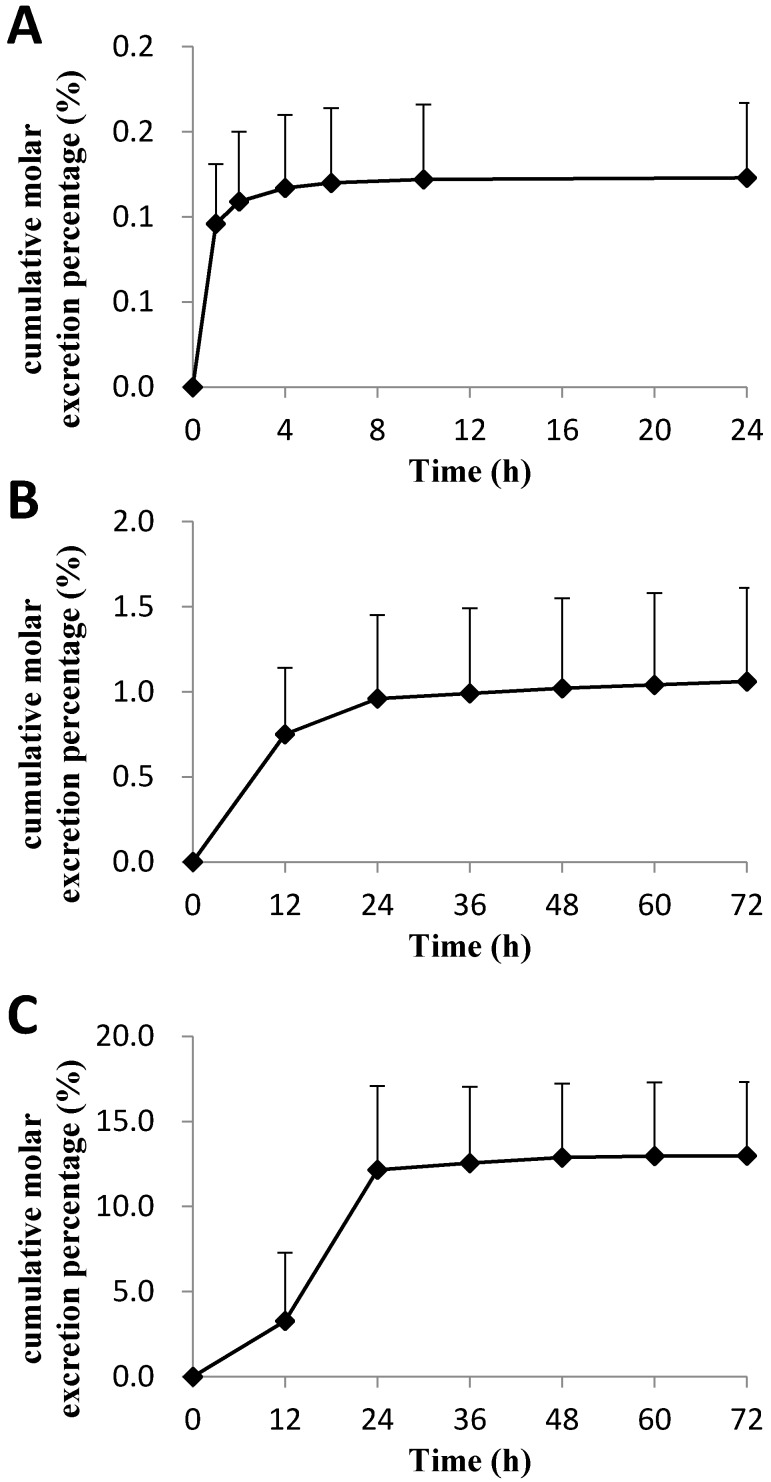
The cumulative molar excretion percentages of C118P and C118 in bile (**A**, *n* = 10), urine (**B**, *n* = 6) and feces (**C**, *n* = 6) of rats after *i.v.* administration of C118P at 20 mg/kg. Data are presented as the arithmetic mean ± SD.

The cumulative molar excretion percentages of C118P and C118 in bile, urine, and feces of rats after intravenous administration of C118P at 20 mg/kg (Figure 4) are 0.123 ± 0.044%, 1.06 ± 0.55%, and 12.98 ± 4.33%, respectively. Further metabolite identification results suggest that the majority of the drugs were excreted in the form of glucuronic acid conjugate and sulfuric acid conjugate, which account for the 42.3 ± 7.3% and 8.8 ± 4.8% excretion of dose in bile, respectively. Glucuronic acid conjugation in urine accounted for the 8.0 ± 2.0% excretion of the dose in 72 h, and no sulfuric acid conjugation was found in urine.

Metabolic stability study showed that C118P is quite stable in liver microsomes from mice, rats, monkeys, and humans. C118 can be metabolized in liver microsomes from different species, with similar ratio of human, mice and SD rats, and very rapid in monkey and very slow in dog. *O*-demethyl, hydroxylated, in the benzyl ring and *N*-oxylation and *N*-acetylation were found. Further glucuronization and sulfation were also observed in the bile excretion.

### 2.6. Discussion

In our preclinical pharmacokinetic study, C118P can be quickly hydrolyzed into C118 after an intravenous administration, and C118 can enter the tissues instantly. The highest tissue concentration of C118 was observed at 10 min after injection. The unbound C118 in plasma is slightly higher than C118P. C118P can hardly penetrate the tissue, while C118 could distribute wildly into tissues. Drug distribution in mice showed that C118 was cleared from the tumor tissue much slower than other tissues, which might be a benefit for antitumor drugs.

We conducted a phase-I and -II metabolic stability assay to predict the in vivo metabolism of C118P and C118. The metabolic clearances, T_1/2_ of C118P, were not calculated because most of the drug remained metabolically intact over 2 h in the live microsome incubation system. The transformation of C118P to C118 is mainly carried out in the blood stream. The rate of biotransformation of C118P to C118 is quite difficult to acquire. The in vitro incubation results showed that C118P is quite stable in mice, rat, monkey, and human microsome incubation systems but not stable in the dog microsome incubation system. The stability study showed that C118 can be rapidly metabolized in the monkey liver microsome incubation system, with a half-life of 2.2 min. C118 is metabolized at the similar speed in mice, rat, and human, with the half-life of 15.5, 10.3, and 11.9 min, respectively. C118 is relatively stable in the dog liver microsome incubation system, with a half-life of 70.1 min. In vivo pharmacokinetic study showed that the mouse plasma half-life (t_1/2_) is longer for C118P than C118. All other animal half-lives show that the half-life (t_1/2_) is shorter for C118P than C118. The (AUC_0-inf_) and C_30s_ of C118P at 50 mg/kg in rats and 6 mg/kg in dogs, and the C_2min_ of C118 at 6 mg/kg in dogs increased less than the dosage increase, suggesting nonlinear pharmacokinetics occurred at high dose. Caution should be paid during the dose scaling during clinical trials, especially at high dose.

When mice were treated with the same dose of C118P or CA4P, the concentration of C118 in plasma, tumor, lung, and muscle is higher than that of CA4 (Table S2). The plasma concentration of C118P is lower than that of CA4P, which suggests that the transformation of C118P into C118 is quicker than CA4P. The concentration of C118 in brain and ovary is lower than that of CA4. Further comparison studies should be carried out in clinical research.

## 3. Materials and Methods

### 3.1. Chemicals and Reagents

C118P standard compound (C118 phosphate, chemical purity is 97.3%), C118P injection (C118P content is 95.6%) and C118 (chemical purity is 95.9%) were manufactured by Nanjing Sanhome Pharmaceutical Co. Ltd. (Nanjing, China). Colchicine (chemical purity is 98.8%, as an internal standard) was purchased from Meryer (Shanghai) Chemical Technology Co., Ltd (Shanghai, China). Methanol and acetonitrile were purchased from Merck (HPLC gradient grade, LiChrosolv GG, Darmstadt, Germany). Milli-Q water was generated from Milli-Q system (Gradient A1, Millipore Inc, Bedford, MA, USA). Pooled human liver microsomes (HLMs) were prepared from eleven Mongolian donors aged from 24 to 38 and were purchased from RILD Research Institute for Liver Diseases Co. Ltd. (Shanghai, China). HPLC grade or the best grade commercially available was used in our study for all other chemicals.

### 3.2. C118P and C118 Analysis in Biological Samples

The concentrations of C118P and C118 in biological samples were determined by HPLC coupled with tandem mass spectrometric detection (LC-MS/MS) after protein precipitation.

The bioanalytical method for simultaneous quantification of C118P and C118 in different matrix was fully validated. Briefly, the plasma, tissue homogenate, thigh bone soak solution, bile, urine, and feces suspension samples were prepared by precipitation with methanol containing internal standard (10 ng/mL). One milliliter of methanol containing internal standard (10 ng/mL) was added to 50 μL of biofluid samples (100 μL for feces suspensions). The mixture was vortexed for 3 min and centrifuged at 18,000 rpm for 5 min. The supernatant was removed and centrifuged again under the same condition. A volume of 200 μL of the second supernatant was transferred into a vial and 5 μL of which was injected into the HPLC-MS/MS system for analysis.

A Shimadzu LC-20A system was equipped with a degasser, binary pump, autosampler, and thermostat-controlled column compartment. Chromatographic separation was performed on an Eclipse XDB-C18 column (4.6 × 150 mm, 5 μm, Agilent) (Santa Clara, CA, USA). Mobile phase was consisted with a mixture of acetonitrile (B)-0.4% formic acid (A) at a flow rate of 0.7 mL/min. The time program of the gradient is described in Table 5. The column temperature was maintained at 40 °C. AB Sciex API 4000 triple quadrupole mass spectrometer (Sciex, Framingham, MA, USA) equipped with an electro-spray-ionization (ESI) Source was used for mass spectrometric detection. The mass spectrometer was operated in the positive mode. The following MS/MS parameters were used: Curtain Gas, 30 Arb; Collision Gas, 10 Pa; Ion Source Gas1, 70 Arb; Ion Source Gas2, 70 Arb; IonSpray Voltage, 5500V; Ion Spray Temperature, 600 °C. Multiple reaction monitoring (MRM) mode was used for mass detection. The optimized MRM fragmentation transitions for C118P was *m*/*z* 407.1 → *m*/*z* 327.1 with a declustering potential of 120 V and Collision Energy of 35 eV and for C118 was *m*/*z* 327.1 → *m*/*z* 312.2 with a declustering potential of 140 V and collision energy of 36 eV. For colchicines (IS), the optimized MRM fragmentation transition was *m*/*z* 400.1 → *m*/*z* 326.1 with a declustering potential of 104 V and collision energy of 36 Ev (Figure S2). The linear range of this analytical method was 5–2000 ng/mL for C118P and 5–500 ng/mL for C118.

### 3.3. Plasma Protein Binding

Plasma protein binding of C118P and C118 were determined by ultrafiltration at four concentrations (C118P: 400, 4000, 40,000, and 400,000 ng/mL, C118: 20, 200, 2000, and 20,000 ng/mL) in pooled plasma from Sprague-Dawley rats, beagle dogs, and humans. Stock solutions of C118P and C118 dissolved in methanol were diluted with blank plasma to achieve the test concentrations (the methanol volume is less than 10% of the plasma). The plasma was incubated for 1 h in a water bath at 37 °C. The total concentration of C118P and C118 of spiked plasma (TC) was analyzed by previously described methods. Ultrafiltration was performed with activated high speed centrifugal tubes (Millipore, Burlington, MA, USA). Plasma samples (0.4 mL) were added to the upper layer of the high speed centrifugal tubes, and then centrifuged at 12,000 g for 10 min. The concentration of unbound C118P and C118 in filtrate (PF) was similarly determined by the previously described methods. The percentage of free fraction (fu) of C118P and C118 was calculated as follows: fu = (PF/TC) × 100%.

### 3.4. Blood Plasma Ratio

The ratio of C118P and C118 in blood and plasma was studied. The whole blood was obtained from healthy volunteer and was separated into two parts to obtain whole blood and plasma. Standard solution was added into whole blood and plasma to mimic the incurred sample. After incubation for 30 min, the whole blood was separated into two parts. One was frozen and thawed to obtain the homogenous whole blood and the other sample was centrifuged to obtain plasma. The samples were analyzed by the established LC-MS/MS method and the blood plasma ratio was calculated.

### 3.5. Metabolic Stability in Liver Microsomes

The in vitro metabolic stability of C118P and C118 were estimated in mice, rat, dog, monkey, and human liver microsomes (Research Institute for Liver Diseases Co. Ltd (Shanghai, China)). The incubation mixture contains C118P or C118, NADP (1.0 mM), G-6-P (10 mM), PDH (2 units/mL), MgCl_2_ (10 mM), and liver microsomes (mice, rats, dogs, monkeys, or humans, 1.0 mg protein/mL) in phosphate buffer. A total of 200 μL of the mixture was removed and quenched with 600 μL acetonitrile in an ice bath at 0, 15, 30, 60, 120, and 240 min after the addition of NADPH.

CL_int, in vitro_ (the in vitro intrinsic clearance, Equation (1)) was calculated from the t_1/2_ of C118 disappearance, where C_protein_ is the protein concentration during the incubation, and t_1/2_ was determined by the slope (k) of the log-linear regression analysis of the concentration versus time profiles; thus, t_1/2_ = ln2/k. The in vivo clearance values (Equation (2)) of mice, rat, dog, monkey, and human were predicted by CL_int, in vitro_ values using physiologically-based scaling factors, hepatic microsomal protein concentrations (47, 47, 58, 32, and 32 mg protein/g liver) [13], and liver weights (54.9, 36.6, 32.9, 24.8 and 25.7 g/kg body weight) [13]. The in vivo hepatic clearance (CL_H_, Equation (3)) was then calculated by using CL_int_ and hepatic blood flow, Q (127,70, 40, 44, and 20 mL·min^−1^·kg^−1^ in rats, dogs, monkey, and humans, respectively) [14,15,16], in the well-stirred liver model disregarding all binding [17]. The hepatic extraction ratio was calculated as CL_H_ divided by Q.

CL_int, in vitro_ = (0.693/t_1/2_) × (1/C_protein_) (1)

CL_int_ = CL_int, in vitro_ × (mg protein/g liver weight) × (gliver weight/kg body weight) (2)

CL_H_ = (Q × CL_int_)/(Q + CL_int_) (3)

### 3.6. PK in Mice, Rats, and Dogs

Experiments in relate to animals were conducted in accordance with the guidelines of Animal Use and Care of the National Institutes of Health and approved by the China Pharmaceutical University Animal Use and Care Committee.

The nude mice (BALB/cA) implanted with NCI-H460 tumor were provided by Shanghai Institute of Materia Medica. A total of 36 mice were divided into 9 groups with four animals in each group. After fasting overnight with free access to water, the mice were treated with 75 mg/kg C118P by *i.v.* The blood samples were collected at 2, 10, 20, 40 min, 1, 2, 4, 8, 12 h after treatment and the mice were anaesthetized and decapitated to obtain the blood and tumor tissue. Blood, brain, muscle, ovary, and lung from mice in the PK study were collected at 10 min, 1, 4, and 12 h. Tissues were cleaned by absorbent filter paper to remove the blood. Bloods were centrifuged at 10,000× *g* for 5 min. Each tissue (0.2 g) was homogenized with 2 mL water. The plasma samples and the homogenates were then processed and determined by the method described above. The concentration was determined by the calibration curve and the actual concentration was corrected by multiplying by 10 according to the dilution times.

The SD rats were supplied by the medical animal experiment center of Chinese People’s Liberation Army Nanjing military region. Twenty-four rats (210–300 g) with half males and half females were divided randomly into four groups. C118P was dissolved in saline to achieve different concentrations for intravenous administration. After fasting overnight, the rats were administered with a single intravenous dose of 5, 10, 20, or 50 mg/kg C118P. Blood samples (approximately 200 μL) were collected in EDTA-coated tubes pre-dose (0), and 30s, 2, 5, 10, 20, 40 min, 1, 2, 4, 8, 12 and 24 h post dose. Plasma was prepared by centrifugation at 8000 rpm for 5 min, and the plasma samples were then prepared and determined by the method described above.

The beagle dogs were purchased from Nanjing Ya Dong experimental animal research center. Twelve beagle dogs with half males and half females were divided randomly into two groups. C118P was dissolved in saline to different concentrations for intravenous administration. After fasting overnight with free access to water, the dogs were administered a single intravenous dose of 1 or 3 mg/kg C118P. After a one-week wash-out period, the group of 1 mg/kg were administered another single intravenous dose of 6 mg/kg C118P. Blood samples (approximately 1 mL) were collected in EDTA-coated tubes at times of 0, 2, 5, 10, 20, 40 min, 1, 2, 4, 8, 12 and 24 h. Plasma was prepared by centrifugation at 8000 rpm for 5 min, and the plasma samples were then prepared and determined by the method described above.

The area under the plasma-concentration-versus-time curve from 0 to infinite (AUC_0-inf_), the elimination half-life (t_1/2_), the systemic plasma clearance (CL) and the volume of distribution (V) were calculated by non-compartmental analysis with WinNonlin software v6.3 (Pharsight Corporation, St. Louis, MO 63144, USA) from the plasma concentration data of C118P and C118, respectively. The instantaneous concentrations after intravenous to rats (C_30s_) or dogs (C_2min_) were obtained directly from the observed data.

Due to the rapid transformation of C118P into C118, there is no direct way to calculate the rate of transformation, thus a two-compartment model with metabolism involved was developed (Figure S3). The transformation rate from C118P into C118 was simulated by fitting the two-serial data of prodrug C118P and C118 in rats and dogs.

### 3.7. Tissue Distribution in Rats

Eighteen rats (weight 220–260 g) with half males and half females were randomly assigned to there groups to carry out a tissue distribution study and fasted overnight. Blood and tissue samples, including the brain, skin, muscle, fat, testicle/ovary, pancreas, spleen, kidney, stomach, small intestine, heart, liver, lung, thymus, and thighbone were collected at 10 min, 1, and 4 h after intravenous administration (20 mg/kg). Blood was centrifuged for plasma samples. Tissue samples were rinsed with water to remove the blood and blotted dry with filter paper. An accurately-weighed amount of the tissue sample (0.2 g) or the entire organ for which weight less than 0.2 g was individually homogenized with EDTA solution (40 mg/mL, 2.0 mL). The left thigh bone was crushed and then soaked overnight in methanol-EDTA solution (1:1, *v*/*v*). A volume of 50 μL of homogenate or soak solution was prepared by protein precipitation and determined by the method described above. Tissue weights were used for calculating the concentrations (ng/g) of C118P and C118 in tissues.

### 3.8. Excretion in Rats

Twelve rats (weight 220–260 g) with half males and half females were anesthetized with diethyl ether after fasted overnight. A median laparotomy was performed and the bile duct was cannulated with a catheter. After intravenous administration (20 mg/kg) of C118P solution, bile samples were collected at 1, 2, 4, 6, 10 and 24 h in EDTA-coated tubes. The accurate volume of bile samples were measured by weight loss method. A volume of 50 μL of bile was prepared by protein precipitation and determined by the method described above. The total cumulative molar excretion percentage of C118P and C118 in bile of ten rats was calculated according to the total molar amount of C118P and C118 and the dose.

Six rats (weight 200–230 g) with half males and half females were fasted overnight. After intravenous administration (20 mg/kg), urine and feces samples were collected at 12, 24, 36, 48, 60 and 72 h. The feces were collected in 200 mL of EDTA solution (100 mg/mL) and grinded into a suspension. The accurately volume amount of urine and feces samples were measured by weight loss method. A volume of 50 μL of urine or feces suspension was prepared by protein precipitation and determined by the method described above. The cumulative molar excretion percentage of C118P and C118 in urine or feces was calculated according to the concentrations of C118P and C118, the volume of each time period.

## 4. Conclusions

In summary, we studied the pharmacokinetic profile of C118P, the prodrug of C118, a novel microtubule protein inhibitor, which is now under clinical trial in China. After *i.v.*, C118P could be hydrolyzed into C118 rapidly, with C118P and C118 detectable in the blood simultaneously. C118 could widely distribute into tissues, with C118P mainly staying in the bloodstream. In tumor-bearing nude mice, the concentration of C118 is high in lung, ovary, and tumor, with an extended half-life in tumors. Since C118 is the active moiety, the PK profile is focused on C118 based on the preclinical study data and its chemistry property. No human pharmacokinetic data could be obtained at the present stage, and the first in-human dosage prediction based on preclinical data could help the phase I clinical trial design and reduce the toxicity risk in humans.

## Figures and Tables

**Table 1:** The fu values of C118P or C118 in rat, dog, and human plasma (%, mean ± SD).

**Species**	**Concentration of C118P (ng/mL)**			
	**400**	**4000**	**40,000**	**400,000**
Rat	3.2 ± 0.1	2.9 ± 0.1	2.4 ± 0.1	12.2 ± 0.6
Dog	1.9 ± 0.1	2.1 ± 0.1	2.7 ± 0.2	8.6 ± 0.3
Human	2.2 ± 0.1	2.7 ± 0.0	2.2 ± 0.1	5.7 ± 0.4
**Species**	**Concentration of C118 (ng/mL)**			
	20 *	**200**	**2000**	**20,000**
Rat	0	7.7 ± 0.8	6.5 ± 0.3	5.5 ± 0.1
Dog	0	5.8 ± 0.2	6.2 ± 0.4	5.4 ± 0.4
Human	0	2.8 ± 0.2	4.7 ± 0.2	3.8 ± 0.2

* The concentrations of C118 in the upper layer were below the lower limit of quantification (LLOQ).

**Table 2:** Metabolic stability of C118 in liver microsomes of different species.

Species	t_1/2_ (min)	CL_int, in vitro_ (μL·min^−1^·mg^−1^ Protein)	CL_int_ (mL·min^−1^·kg^−1^)	Extrapolated CL_H_ (mL·min^−1^·kg^−1^)	Observed CL (mL·min^−1^·kg^−1^)
Mice	15.5	44.7	115.3	49.3	88.8
Rat	10.3	67.0	115.3	43.6	62.9
Dog	70.1	9.9	18.9	12.8	54.0
Monkey	2.2	318.6	252.8	37.5	/
Human	11.9	58.2	47.9	14.1	/

**Table 3:** Pharmacokinetic parameters of C118P and C118 in nude mice, rats and dogs after intravenous injection of C118P (*n* = 4 for nude mice and *n* = 6 for rats and dogs, mean ± SD).

Species	Dose (mg/kg)		Compound	T_1/2_ (h)	C_2min_ (ng/mL, ng/g) *	AUC_0-inf_ (ng × h/mL, ng × h/g)	V (mL/kg, g/kg)	CL (mL/h/kg, g/h/kg)
mice	75	plasma	C118P	1.96 ± 1.07	105,685.1 ± 29,837.1	13,523.8 ± 3444.3	15,404.8 ± 7499.6	5812.5 ± 1484.3
			C118	0.97 ± 0.10	42,476.2 ± 8033.0	13,371.6 ± 1588.1	/	/
		tumor	C118P	1.16 ± 0.76	291.6 ± 80.2	217.7 ± 9.3	/	/
			C118	3.70 ± 1.48	13,757.4 ± 2475.6	73,027.8 ± 24,951.2	/	/
rats	5	plasma	C118P	0.24 ± 0.05	75,624.6 ± 9297.9	5740.3 ± 765.5	302.5 ± 61.1	882.9 ± 116.9
			C118	0.52 ± 0.10	1578.4 ± 318.1	740.7 ± 63.2	/	/
	10	plasma	C118P	0.21 ± 0.05	133,672.5 ± 17,831.1	10,446.7 ± 1898.9	299.4 ± 67.6	984.6 ± 186.3
			C118	0.55 ± 0.13	3720.8 ± 973.8	1598.8 ± 192.9	/	/
	20	plasma	C118P	0.38 ± 0.10	219,293.0 ± 49,528.2	18,743.7 ± 3511.1	582.6 ± 114.2	1108.0 ± 272.9
			C118	0.60 ± 0.06	7716.7 ± 2005.6	3751.1 ± 592.5	/	/
	50	plasma	C118P	0.80 ± 0.57	372,898.3 ± 28,583.8	38,877.1 ± 8164.2	1502.2 ± 1065.8	1338.6 ± 324.4
			C118	2.49 ± 1.26	20,590.0 ± 6376.3	9723.1 ± 1298.0	/	/
dogs	1	plasma	C118P	0.22 ± 0.09	12,260.2 ± 3233.4	1696.2 ± 345.9	181.7 ± 51.3	611.5 ± 145.1
			C118	0.49 ± 0.18	252.1 ± 70.5	214.3 ± 79.0	/	/
	3	plasma	C118P	0.59 ± 0.16	34,685.8 ± 7469.8	5301.0 ± 1698.4	509.2 ± 162.0	606.5 ± 163.0
			C118	1.05 ± 0.27	860.9 ± 258.9	910.0 ± 294.8	/	/
	6	plasma	C118P	0.39 ± 0.12	64,181.4 ± 16,257.0	8530.5 ± 1549.4	398.5 ± 87.1	726.1 ± 159.0
			C118	0.84 ± 0.25	1702.7 ± 508.6	1829.6 ± 320.4	/	/

*: C_30s_ for Rat.

**Table 4:** The distribution of C118P and C118 in tumor bearing mice after *i.v.* injection of 75 mg/kg of C118P (*n* = 4, mean ± SD).

Tissue	Time (h)	C118P	C118
Plasma	0.17	5199.4 ± 2147.9 *	26,048.0 ± 2429.7 ^##^
	1	191.2 ± 60.5	1730.0 ± 986.4
	4	14.7 ± 8.4	132.6 ± 56.8
	12	6.1 ± 0.0	NF
Tumor	0.17	115.8 ± 24.1	14,563.3 ± 1989.9 ^#^
	1	73.0 ± 15.0	12,998.1 ± 4132.3
	4	26.7 ± 0.0	4969.8 ± 3414.7
	12	NF	1327.7 ± 1124.0
Brain	0.17	NF	3562.8 ± 796.6 ^##^
	1	NF	1250.7 ± 351.3
	4	NF	109.1 ± 22.0
	12	NF	NF
Lung	0.17	130.2 ± 7.5	26,923.2 ± 4389.0 ^##^
	1	NF	1852.3 ± 929.3
	4	NF	231.3 ± 181.7
	12	NF	NF
Ovary	0.17	NF	26,723.8 ± 18,229.6
	1	NF	3308.6 ± 2611.1
	4	NF	NF
	12	NF	NF
Muscle	0.17	355.4 ± 64.1	15,202.5 ± 8814.7
	1	NF	946.8 ± 390.6
	4	NF	136.4 ± 2.8
	12	NF	NF

NF: not found, *: compared with data from CA4P (*p* < 0.05) in supplemental data (Table S2); ^#^: compared with data from CA4 (*p* < 0.05) in supplemental data (Table S2); ^##^: compared with data from CA4 (*p* < 0.01) in supplemental data (Table S2).

**Table 5:** Gradient program for HPLC separation of C118P and C118.

Time (min)	Mobile Phase	
	A (%)	B (%)
0	95	5
1	95	5
5.5	40	60
7	2	98
7.5	2	98
7.51	95	5
12	95	5

Solvent A: 0.4% formic acid. Solvent B: acetonitrile.

## Supplementary Materials

The following are available online, Figure S1: (**A**) Concentration-time curves of C118P after C118P incubation with mice, rat, dog, monkey and human liver microsomes (ng/mL); (**B**) Concentration-time curves of C118 after C118P incubation with mice, rat, dog, monkey and human liver microsomes (ng/mL); Figure S2: The mass spectrum of C118P (A), C118 (B) and internal standard colchicines (C); Figure S3: The pharmacokinetic model of C118P and C118 in rat after *i.v.* C118P; Figure S4: The simulation of PK profile at 20 mg/kg in rat (A) and 6 mg/kg (B) in dog based on initial parameters calculated by Winnonlin; Table S1: The initial parameters and simulated parameters of C118P and C118 used in the tandem two-compartmental model; Table S2: The distribution of CA4P and CA4 in tumor bearing mice after *i.v.* injection of 75 mg/kg of CA4P.
