# Supplementary material for: Preclinical Pharmacokinetics of C118P, a Novel Prodrug of Microtubules Inhibitor and Its Metabolite C118 in Mice, Rats, and Dogs
Source: Molecules. 2018 Nov 5;23(11):2883. doi: 10.3390/molecules23112883 (PMC6278385; doi:10.3390/molecules23112883)
Supplement: Supplementary file 1 [file molecules-23-02883-s001.pdf]

Supplementary Materials

1. The *in vitro* metabolic stability of C118P and C118 were estimated in mice, rat, dog, monkey and human liver microsomes. Results showed that C118P was quite stable in liver microsomes of human, mice, rat and monkey and is not stable in dog microsome (**Figure S1**).

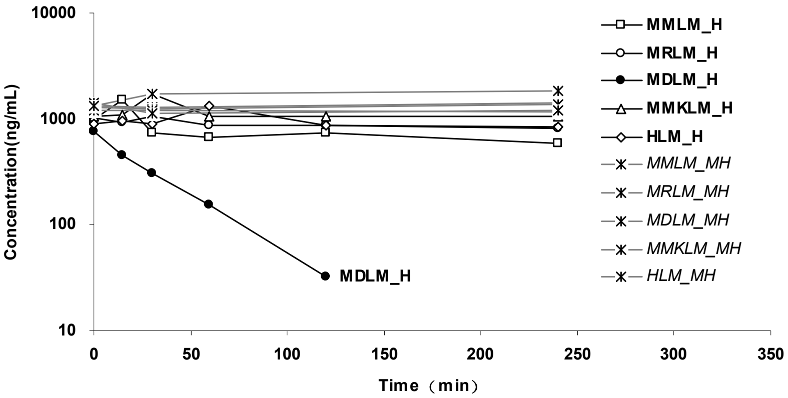

Figure S1A. Concentration-time curves of C118P after C118P incubation with mice, rat, dog, monkey and human liver microsomes (ng/ml), where _H represents the active microsome group and _MH represents the deactivated microsome group.

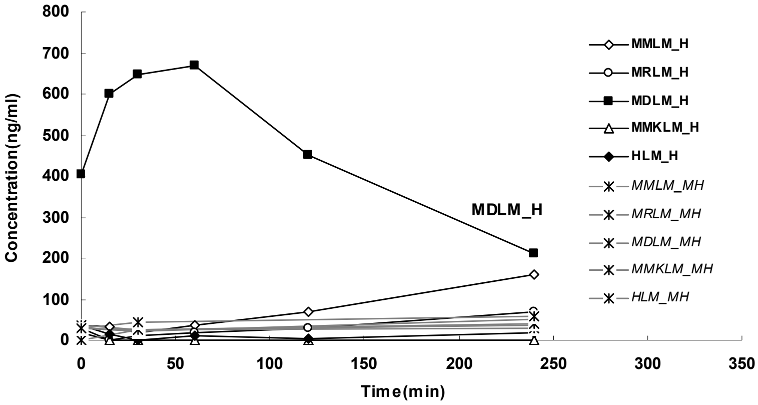

Figure S1B. Concentration-time curves of C118 after C118P incubation with mice, rat, dog, monkey and human liver microsomes (ng/ml) , where _H represents the active microsome group and _MH represents the deactivated microsome group.

2. During method development, the mass spectrometer was operated in the positive mode. The optimized MRM fragmentation transitions for C118P was m/z 407.1→ m/z 327.1 (**Figure S2A**)and for C118 (**Figure S2B**)was m/z 327.1→ m/z 312.2. For colchicines (IS), the optimized MRM

fragmentation transition was m/z 400.1→ m/z 326.1(**Figure S2C**).

A

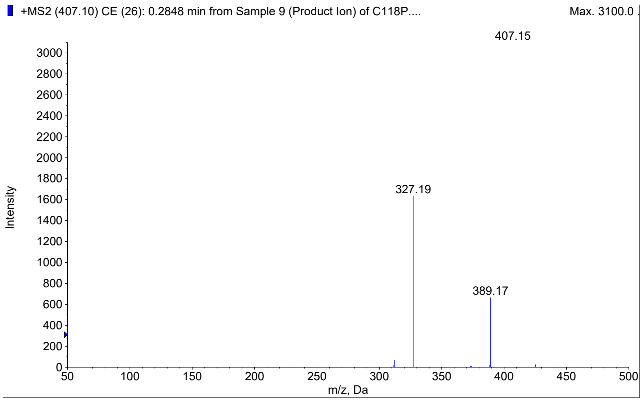

B

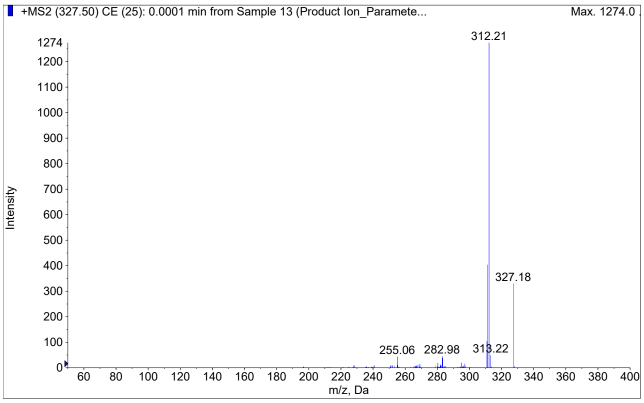

C

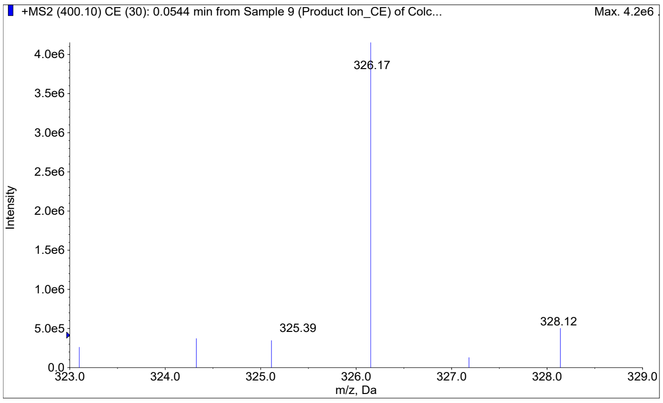

Figure S2. The mass spectrum of C118P(A), C118(B) and internal standard colchicines(C)

3. The pharmacokinetic profile of C118P and C118 was simulated by two compartmental models. To simplify the model, the excretion rate of C118P from central compartment was supposed to be zero. In this model, both C118P and C118 were described by two-compartmental model, with elimination rate of k(0,3) for C118. The connection between C118P and C118 is by the uniflow of transformation rate of k(3,1) since the metabolism of C118P to C118 is irreversible(**Figure S3**).

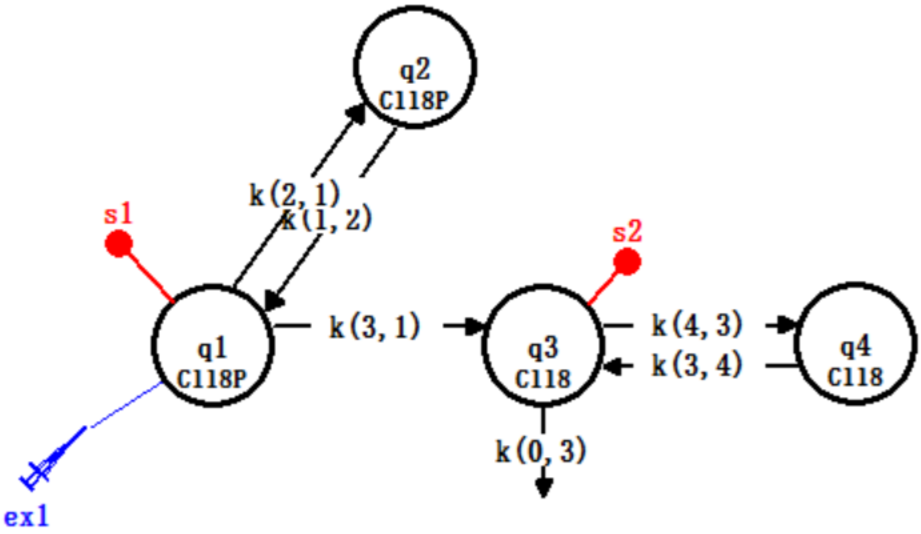

Figure S3. The pharmacokinetic model of C118P and C118 in rat after i.v C118P. ex1: the injection dose of C118P, s1: concentration of C118 in central compartment. s2: concentration of C118 in central compartment, q1 and q3 represent the central compartment of C118P and C118, respectively. The q2 and q4 represent the peripheral compartment of C118P and C118, respectively. k(0,3) represents the elimination rate of C118 from central compartment. k(3,1) represents the transformation rate of C118P to C118. k(2,1) and k(4,3) represent the distribution rate of C118P and C118 from central compartment to peripheral compartment. k(1,2) and k(3,4) represent the return rate of C118P and C118 from peripheral compartment to central compartment.

For prodrug pharmacokinetic prediction, the transformation of prodrug to the active drug should be studied in different system to get the right data. With the help of modeling, the transformation ratio of C118P to C118 was calculated by a tandem two-compartment model (SAAMII, University of Washington). In simulation study, the initial parameters are very important for accurate prediction. The initial compartmental model parameters of rat and dog were first evaluated by Winnonlin using two-compartmental model at low dose, with intravenous for C118P and extra vessel for C118 since

C118 was transformed from C118P. The initial parameters used in the simulation were listed in **Table S1**. Using these parameters, the simulation of PK profiles of rat and dog are well fit to the data from in vivo at higher dose **(Figure S4)**. The simulation of C118P and C118, the transformation rate of C118P to C118 is calculated and transformation ratio (k) is about 18.3671/h and12.588 1/h for rats and dogs, respectively.

A

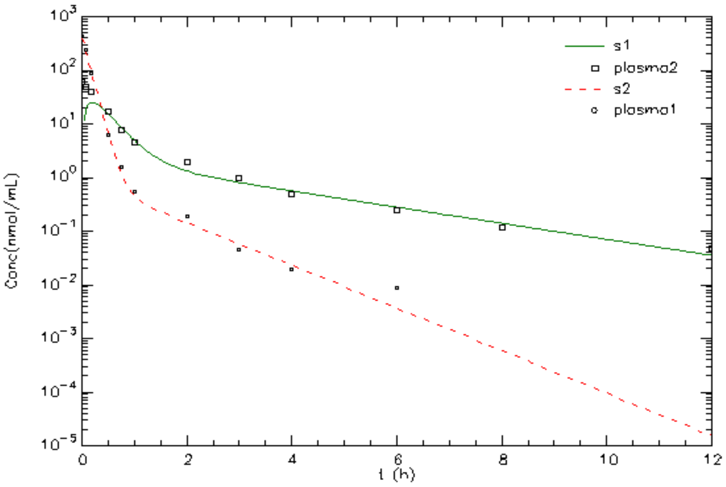

B

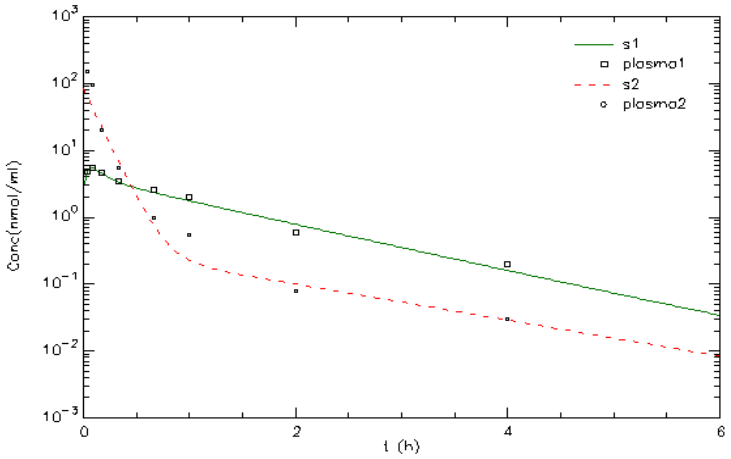

Figure S4. The simulation of PK profile at 20 mg/kg in rat(A) and 6 mg/kg(B) in dog based on initial parameters calculated by Winnonlin. (s1 represent the simulated data for C118 and s2 represent the simulated data for C118P, plasma 1 represent the experimental data for C118 and plasma 2 represent the experimental data for C118P.)

Table S1. The initial parameters and simulated parameters of C118P and C118 used in the tandem two-compartmental model.

Parameter	Units	rat		dog	
		C118P	C118	C118P	C118
V1	mL/kg	54.41	2708	50.97	220.5
K10*	1/h	18.369	0.9897	12.558	3.1444
K12	1/h	6.535	0.3516	1.844	1.3247
K21	1/h	18.281	0.0121	4.727	0.2110

K10: is supposed to be the transformation rate of C118P into C118 and was simulated based on the fixed parameters.

4. The distribution of CA4P and CA4 in tumor bearing mice.

The nude mice (BALB/cA) implanted with NCI-H460 tumor were provided by Shanghai institute of Materia Medica. A total of 36 mice were divided into 9 groups with 4 animals in each group. After fasted overnight with free access to water, the mice were treated with 75 mg/kg CA4P by *i.v.* The blood samples were collected at 2, 10, 20, 40 min, 1, 2, 4, 8, 12 h after treatment and the mice were anaesthetized and decapitated to obtain the blood and tumor tissue. All procedure are same as C118P in the main text. The concentration was determined by the calibration curve and the actual concentration was corrected by multiplying by 10 according to the dilution times.

Table S2 The distribution of CA4P and CA4 in tumor bearing mice after i.v injection of 75 mg/kg of CA4P（n=4，Mean±SD）

tissue	Time（h）	CA4P	CA4
Plasma	0.17	15879.9±4473.5	12540.3±972.6
	1	324.9±105.2	595.8±277.7
	4	15.0±6.8	21.1±4.5
	12	12.6±3.3	NF
Tumor	0.17	142.8±49.2	9835.7±1345.9
	1	26.5±1.4	4803.9±770.6
	4	34.5±1.9	3677.0±1079.9
	12	NF	735.1±426.2
brain	0.17	NF	16165.3±1211.9
	1	NF	2135.9±486.2
	4	NF	97.6±21.3
	12	NF	NF
lung	0.17	NF	5849.0±580.7
	1	NF	759.4±242.6
	4	NF	106.8±12.1
	12	NF	NF
ovary	0.17	NF	44183.5±13169.9
	1	NF	1288.3±711.2
	4	NF	NF
	12	NF	NF
muscle	0.17	345.2±68.9	8526.1±3505.7
	1	NF	595.6±308.4
	4	NF	142.0±33.9
	12	NF	51.9±36.8

NF:not found
